# The embodied typist: Bimanual actions are modulated by words’ implied motility and number of evoked limbs

**DOI:** 10.1371/journal.pone.0289926

**Published:** 2023-08-10

**Authors:** Katia Rolán, Iván Sánchez-Borges, Boris Kogan, Enrique García-Marco, Carlos J. Álvarez, Manuel de Vega, Adolfo M. García

**Affiliations:** 1 Instituto Universitario de Neurociencia, Universidad de La Laguna, La Laguna, Spain; 2 Laboratorio de Linguaxe e Cognición, Universidade de Vigo, Vigo, Spain; 3 Departamento de Filosofía, Facultad de Humanidades, Universidad Nacional de Mar del Plata, Buenos Aires, Argentina; 4 Consejo Nacional de Investigaciones Científicas y Técnicas, Buenos Aires, Argentina; 5 Centro de Neurociencias Cognitivas, Universidad de San Andrés, Buenos Aires, Argentina; 6 Departamento de Psicología Clínica y Experimental, Universidad de Huelva, Huelva, Spain; 7 Global Brain Health Institute, University of California, San Francisco, San Francisco, California, United States of America; 8 Departamento de Lingüística y Literatura, Facultad de Humanidades, Universidad de Santiago de Chile, Santiago, Chile; Universita di Bologna, ITALY

## Abstract

The planning and execution of manual actions can be influenced by concomitant processing of manual action verbs. However, this phenomenon manifests in varied ways throughout the literature, ranging from facilitation to interference effects. Suggestively, stimuli across studies vary randomly in two potentially relevant variables: verb motility and effector quantity (i.e., the amount of movement and the number of hands implied by the word, respectively). Here we examine the role of these factors during keyboard typing, a strategic bimanual task validated in previous works. Forty-one participants read and typed high and low motility items from four categories: bimanual, unimanual, and non-manual action verbs, as well as minimally motoric verbs. Motor planning and execution were captured by first-letter lag (the lapse between word presentation and first keystroke) and whole-word lag (the lapse between the first and last keystroke). We found that verb motility modulated action planning and execution, both stages being delayed by high (relative to low) motility verbs. Effector quantity also influenced both stages, which were facilitated by bimanual verbs relative to unimanual verbs and non-manual verbs (this effect being confined to high motility items during action execution). Accordingly, motor-language coupling effects seem sensitive to words’ implied motility and number of evoked limbs. These findings refine our understanding of how semantics influences bodily movement.

## Introduction

Research on motor-language coupling has revealed direct links between lexico-semantic processing and physical action, especially for the domain of manual action verbs [1–7]. Yet, depending on stimulus- and task-related factors, relevant studies have shown facilitation, interference, and null effects [1, 8], calling for new insights on the phenomenon. Suggestively, most experiments employ word lists that vary randomly in terms of motility (the amount of movement involved, from high to low) and effector quantity (mixing bimanual and unimanual actions). To disentangle the role of both factors in motor-language coupling, we asked participants to perform a bimanual motor task (keyboard typing) as they processed high and low motility items from four categories: bimanual, unimanual, and non-manual, as well as minimally motoric verbs. Importantly, this task has been shown to capture robust motor-language coupling effects with different stimuli both during action planning and execution [9–11].

Motor-language coupling is the embodied, context-sensitive phenomenon whereby action-laden words influence bodily movements, and vice versa [1, 2, 6, 12–15]. These effects can manifest in an effector-specific fashion, such that, for instance, words evoking a specific limb can distinctly affect movements of that body part [9, 10, 12]. However, the direction of this influence varies substantially across studies, as seen in research combining manual verbs with manual actions.

Some studies have found facilitation effects, such as faster manual responses to manual verbs after viewing congruent action videos [16] or following stimulation of arm/hand brain circuits [4]. Suggestively, specific manual actions (e.g., object grasping and displacement) can be distinctly facilitated when manual verbs evoke congruent movements [17]. Yet, other experiments have yielded interference effects, including slower hand movements during semantic decision [18] and congruency judgment [19] tasks. Still other works have reported mixed effects for manual verbs, such as faster initiation but slower execution of manual actions [3, 20, 21] or selective acceleration of execution (as opposed to initiation) routines during word writing [12]. These varying results likely reflect task- and stimulus-related discrepancies across studies, calling for more nuanced designs [1]. To tackle this challenge, we target here two potentially critical yet overlooked factors: verb motility and effector quantity–i.e., the amount of movement and the number of hands implied by the verb’s meaning, respectively.

First, motor-language coupling might be sensitive to the task’s implied motility–namely, the amount of movement evoked by the action verbs or involved in the participants’ response. For example, manual verbs, at large, are processed less efficiently when accompanied by fast manual actions [22], and those evoking fast (as opposed to slow) actions are distinctly impaired in patients with motor-system damage [23]. More particularly, high motility verbs are selectively compromised in persons with movement disorders and preserved cognitive skills [24]. Motor mechanisms, then, might be distinctly taxed by processes entailing high motility, leading to behavioral interference. Indeed, motor-region activity increases in tasks with high motor demands [25], potentially consuming resources required for other action-related processes [1, 26]. Altogether, this motivates our study’s first question: might motor-language coupling be influenced by verbs’ implied motility?.

Second, the phenomenon may also be influenced by congruency between the limbs evoked by the verb and employed to respond. In particular, muscle activation induced by manual verbs proves faster in both hands during bimanual compared to unimanual tasks [27]. Compatibly, while unimanual verbs engage only left-sided motor regions, bimanual verbs typically produce a bilateral motor cortex activation [28]. Accordingly, bimanual activities could be distinctly primed by words evoking two-hand actions. This aligns with evidence showing that specific actions may be facilitated by words that prime relevant movement features [1] and that bimanual coordination can be influenced by linguistic content [7]. Such antecedents prompt our second question: are bimanual actions distinctly affected by manual verbs that evoke motoric activity of both hands?.

Despite the paucity of direct evidence, both factors are contemplated by an explicit theoretical framework: the Hand-Action-Network Dynamic Language Embodiment (HANDLE) model [1]. Integrating network activation and predictive coding principles, HANDLE identifies conditions under which manual verbs would either delay or facilitate manual behavior. First, HANDLE posits that when hand-specific motor networks are taxed by a given process (e.g., verb comprehension), they become suboptimally available for subsequent processes (e.g., manual actions), leading to behavioral interference. Therefore, if verb motility is associated with motor-system recruitment, then high motility manual verbs should delay ensuing hand movements. Second, drawing from predictive coding principles [29], HANDLE proposes that effector-specific semantic information prompts predictions which may or not be satisfied by a subsequent motoric process. Upon processing a bimanual verbs, then, error correction demands would be lower for bimanual than unimanual actions, such that the former would become facilitated. These tenets provide a rationale for disentangling the role of verb motility and effector quantity during motor-language coupling, while favoring their integration with an overarching account of the topic.

Against this background, we examined the impact of verb motility and effector quantity on motor-language coupling. Strategically, we leveraged an ecological keyboard-based verb copying task, which integrates linguistic (verb reading) and motoric (key-pressing) processes as participants plan and execute two-handed actions (typing). Our design involved four verb categories (bimanual, unimanual, non-manual, and minimally motoric verbs), each comprising high and low motility items. As in previous reports of this paradigm [9–11], we examined the effect of such factors on both motor planning and execution. We predicted that, relative to low motility verbs, high motility verbs would involve longer planning and execution stages. Also, considering that typing is a bimanual activity, we hypothesized that both stages would prove faster for bimanual verbs than for other verb categories. Moreover, given our goal to disentangle both factors and account for their interplay in terms of HANDLE, we explored their possible interactions through a factorial design. Briefly, this approach aims to illuminate key factors shaping the integration of verbal and motoric information.

## Materials and methods

### Participants

We recruited 41 participants, reaching a power of .97 (Section 1 in S1 File). The sample comprised right-handed Spanish-speaking individuals with normal or corrected-to-normal vision and a mean of 21.1 years of age (*SD* = 7.1). Information about computer-related knowledge and experience was collected through a previously reported questionnaire [10, 11] with five-point Likert-type scales (1 = null, 2 = low, 3 = medium, 4 = advanced, 5 = expert). The group’s operational knowledge fell between intermediate and advanced in terms of hardware (*M* = 3.17, *SD* = 0.74) and software (*M* = 3.15, *SD* = 0.64) skills. Most participants (90%) were frequent Windows users, while the other 10% mainly used Mac computers. All participants rated their general typing skills between intermediate and advanced (*M* = 3.6, *SD* = 0.82), using a mean of 7.4 (*SD* = 2.5) fingers for the task. As regards gaze habits during typing, 18 participants stated focusing mainly on the screen, 10 looked at the keyboard, and the rest reported similar gaze distribution between the screen and the keyboard. All participants provided written informed consent in accordance with the Declaration of Helsinki. The study was approved by the ethics’ committee of Universidad de La Laguna.

### Stimuli

Stimuli consisted of short Spanish sentences in present continuous tense, starting with *Estás* (*You are*) and followed by a target verb (e.g., *cosiendo* [*sewing*]). The target verbs comprised 208 items from four categories: bimanual verbs (*N* = 52), denoting actions performed with two hands (e.g., *aplaudiendo* [*clapping*]); unimanual verbs (*N* = 52), denoting actions performed with one hand (e.g., *firmando* [*signing*]); non-manual verbs (*N* = 52), denoting actions performed with body parts other than the hands (e.g., *caminando* [*walking*]); and minimally motoric verbs (*N* = 52), denoting little or no motion (e.g., *amando* [*loving*]). Minimally motoric verbs were included as a benchmark condition involving little to no sensorimotor resonance [12, 26]. Each set comprised 26 high motility and 26 low motility items, based on median splits of their normative motility ratings [30]. Crucially, motility was significantly higher for the high than the low motility items in each verb type (all *p*-values > 0.01)–see S1 Table in S2 File.

Stimuli were selected following validated protocols for keyboard typing paradigms [10, 11]. Specifically, comparability among conditions was confirmed via pairwise comparisons across all verb types and motility levels. Across all four verbs types, high and low motility items were matched for frequency, number of letters, number of syllables, orthographic neighbors, phonological neighbors, familiarity, imageability and concreteness–based on normative data from EsPal Database [31]–, as well as age of acquisition–based on normative data [30]. Moreover, their typing required similar numbers of strokes in six areas of QWERTY keyboards (qwert, asdfg, zxcv, yuiop, hjkl, bnm). Crucially, high motility verbs had similar motility ratings in all action verb categories (bimanual, unimanual, and non-manual), which was also true for low motility verbs. As expected, motility, imageability, and concreteness ratings were lower for minimally motoric verbs than for the three action categories. For the full stimulus list (including approximate English translations) and statistical details, see S1-S8 Tables in S2 File, S9 Table in S3 File.

### Design and procedure

Participants were evaluated individually in a quiet room, where they sat comfortably at a desk. They faced a laptop equipped with a 24” 16:9 HD (1366 x 768) LED backlight display and a QWERTY keyboard including Spanish characters. In each trial, participants were presented with a brief grammatical context (*Estás* [*You are*]) followed by a target verb as described in the Materials section (e.g., *aplaudiendo* [*clapping*]). They were instructed to type the target verb as fast and accurately as possible in a single uninterrupted action. They were further told to press the spacebar after typing was complete, in order to launch the following trial. Eight practice trials were presented at the beginning for familiarization purposes. Stimuli from the four categories were pseudorandomly distributed across four blocks of 52 trials. A brief break was allowed between blocks. The task involved a 2x4 design, with motility as a two-level factor (high, low) and verb type as a four-level factor (bimanual, unimanual, non-manual, and minimally motoric verbs).

**Fig 1:**
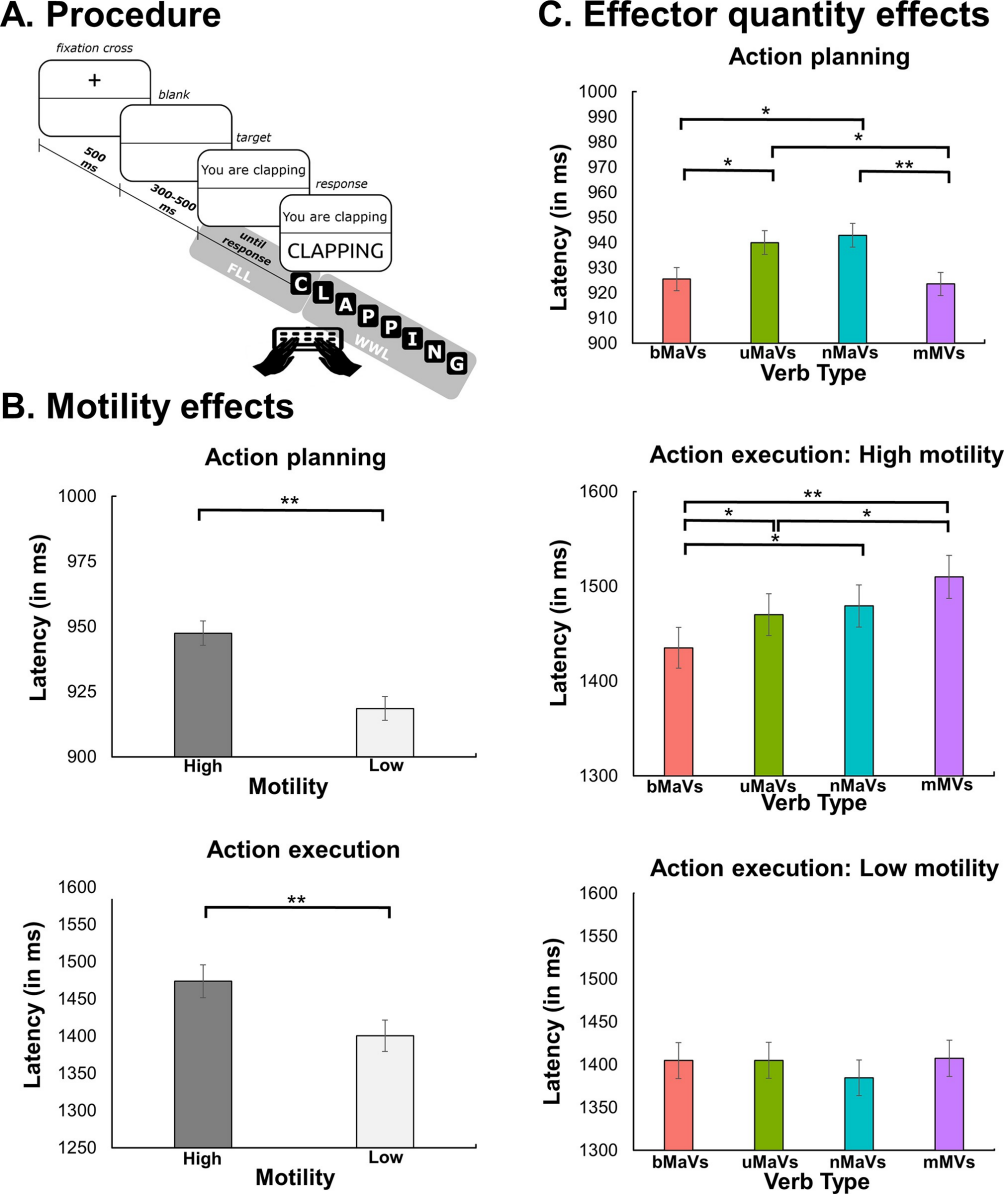
Procedure and results. **(A)** Structure of the verb-copying task. Participants were instructed to type the target verb as fast and accurately as possible in a single uninterrupted gesture. The figure illustrates a single trial from the bimanual verb condition. **(B)** Motility effects: with significantly longer latencies for high motility verbs compared to low motility verbs, both in action planning (indexed by first-letter lag) and action execution (indexed by whole-word lag). **(C)** Effector quantity effects: Action planning, with significantly shorter latencies for bimanual compared to unimanual and non-manual verbs; and for unimanual and non-manual compared to minimally motoric verbs; and Action execution, with a significant interaction between motility and verb type. In the high motility condition, bimanual verbs were faster than unimanual, non-manual, and minimally motoric verbs. Also, unimanual verbs were faster than minimally motoric verbs. The low motility condition revealed no significant differences between verb types. Single asterisks (*) indicate a statistically significant difference at *p* < .05. Double asterisks (**) indicate a statistically significant difference at *p* < .01. FLL: first-letter lag (lapse between target onset and first keystroke). WWL: whole-word lag (lapse between first and last keystroke).

Each trial began with an ocular fixation cross at the center of the screen. The verb remained on the screen until the participant gave a complete response. The fixation cross and the targets (font: Courier New; color: black; size: 18; style: regular) were presented in the middle of a grey panel occupying the upper half of the screen. Pressing the spacebar after the target was copied triggered the following trial. Trial-onset asynchrony randomly varied between 300 and 500 ms, to minimize the predictability of the target. The paradigm was designed and run on E-Prime software 2.0 (Psychology Software Tools, Pittsburgh, PA). The complete session lasted roughly 25 min. For a detailed structure of a single trial, see Fig 1A.

As in previous keylogging studies [9–11], we considered three dependent variables. Motor programming was indexed by the first-letter lag (FLL) measure, defined as the time-lapse between word presentation and the first keystroke made thereon. Motor execution was operationalized as whole-word lag (WWL), namely, the time-lapse between the first and last keystroke on a trial–prior to a spacebar press for launching the following trial. Accuracy was assessed in terms of failed typing responses, so that a trial was considered incorrect if its keyboard sequence included a typo and/or missing or added characters (note that the ‘delete’ key was disabled during the task).

### Statistical analysis

Analyses were based on a 2x4 repeated measures design with the factors motility (high, low) and verb type (bimanual, unimanual, non-manual, and minimally motoric verbs). Data removal criteria were adopted from previous keylogging research [11]. The E-Prime script automatically calculated FLL and WWL for each trial. Within each condition, failed typing responses were excluded from FLL and WWL analyses. Then, responses were further rejected if they exceeded 2.5 *SDs* from the participant’s mean in each measure and condition (rejected trials amounted to 2.54% for FLL and 1.89% for WWL. The 2x4 ANOVAs were run on the remaining FLL and WWL data. Hochberg´s post hoc test was used to examine pairwise comparisons for significant effects of verb type and significant interactions. In all cases, alpha levels were set at .05. Effect sizes for main effects were calculated with partial eta squared ($\eta_{p}^{2}$), ranging from small (> .02) to medium (> .13) to large (> .26) [32]. Given the small effect sizes of motor-language coupling phenomena [1, 33] and the adequate power of our sample, post hoc comparisons were performed without correcting for multiple comparisons, thus reducing the likelihood of Type II errors. Effect sizes for pairwise comparisons were calculated through Cohen’s *d* [32], an index that discriminates among small (0–0.20), medium (0.50–0.80), and large (> 0.80) effects [32]. Analyses were performed on R software (version 3.4.0), by means of the ULLRToolbox (https://sites.google.com/site/ullrtoolbox/home).

## Results

### Accuracy

The total average number of failed typing responses was 17.3%. There were no significant differences between high (*M* = 82.2%, *SD* = 11.5%) and low (*M* = 83.1%, *SD* = 11.7%) motility verbs [*F* (1, 39) = 1.53, *p* = .22, $\eta_{p}^{2}$ = .04]), nor among verb types (bimanual verbs: *M* = 82.4% *SD* = 12.3%; unimanual verbs: *M* = 82.54%, *SD* = 11.9%; non-manual verbs: *M* = 82.3%, *SD* = 12.1; minimally motoric verbs: *M* = 83.2%, *SD* = 10.0%) [*F*(3, 117) = .27, *p* = .84, $\eta_{p}^{2}$ = .03]. The interaction between both factors was also non-significant [*F*(3, 117) = 1.31, *p* = .27, $\eta_{p}^{2}$ = .08]. For details, see Section 3 in S3 File.

### FLL results

FLL outcomes showed a significant effect of motility, with longer latencies for high (*M* = 947 ms, *SD* = 218 ms) than low (*M* = 919 ms, *SD* = 214 ms) motility verbs [*F*(1, 39) = 21.77, *p* < .001, $\eta_{p}^{2}$ = .36])–Fig 1B, top panel. The effect of verb type was also significant [*F*(3, 117) = 4.21, *p* < .01, $\eta_{p}^{2}$ = .25], with lower latencies for bimanual verbs (*M* = 925 ms *SD* = 228) than unimanual verbs (*M* = 940 ms, *SD* = 216 ms) (estimate = -14.5, *t*(273) = 2.03, *p* < .05, *d* = .07) and non-manual verbs (*M* = 942 ms, *SD* = 210 ms) (estimate = -17.4, *t*(273) = 2.44, *p* < .05, *d* = .08); and for minimally motoric verbs (*M* = 924 ms, *SD* = 213 ms) than unimanual verbs (estimate = -16.5, *t*(273) = 2.30, *p* < .05, *d* = .08) and non-manual verbs (estimate = -19.3, *t*(273) = 2.70, *p* < .01, *d* = .07)–Fig 1C, top panel. No other significant differences were found between verb types (all *p-*values > .19). The interaction between motility and verb type was not significant [*F*(3, 117) = 1.18, *p* = .32, $\eta_{p}^{2}$ = .08]. For details, see Section 3 in S3 File.

### WWL results

WWL results yielded a significant effect of motility [*F*(1, 39) = 70.74, *p* < .001, $\eta_{p}^{2}$ = .64)], with longer latencies for high (*M =* 1474 ms; *SD* = 311) than low (*M =* 1400 ms; *SD* = 301) motility verbs–Fig 1B, bottom panel. We also found a main effect of verb type [*F*(3, 117) = 3.47, *p* < .05, $\eta_{p}^{2}$ = .24], mediated by motility, as seen in the significant interaction between both factors [*F*(3, 117) = 3.43, *p* < .02, $\eta_{p}^{2}$ = .23]. Post-hoc contrasts revealed that high motility items yielded significantly different WWLs among verb types (Fig 1C, middle panel), with bimanual verbs (*M =* 1435 ms; *SD* = 294 ms) yielding faster responses than unimanual verbs (*M =* 1470 ms; *SD* = 314 ms) (estimate = -35, *t*(273) = 2.00, *p* < .05, *d* = .02), non-manual verbs (*M =* 1479 ms; *SD* = 316 ms) (estimate = -44, *t*(273) = 2.53, *p* < .05, *d* = .01), and minimally motoric verbs (*M =* 1510 ms; *SD* = 327) (estimate = -75, *t*(273) = 4.27, *p* < .001, *d* = .01). In addition, unimanual verbs were faster than minimally motoric verbs (estimate = -40, *t*(273) = 2.27, *p* < .05, *d* = .01). Conversely, no significant differences among verb types with low motility (all *p*-values > .19)–Fig 1C, bottom panel. No other significant differences arose (all *p-*values > .08). For details, see Section 3 in S3 File.

## Discussion

We aimed to disentangle the role of verbs’ implied motility and effector quantity during motor-language coupling. Motility modulated action planning and execution, both stages being delayed by high (relative to low) motility verbs. Effector quantity also modulated both stages, which were facilitated by bimanual verbs relative to unimanual verbs and non-manual verbs (this effect being confined to high motility items during action execution). Such results shed new light on how semantics influences bodily movement, as described below.

The planning and execution of typing routines were delayed by high (relative to low) motility verbs. This indicates that the integration of semantic and motoric processes is sensitive to the task’s implied action load. Compatible findings were reported by Speed and colleagues [22], who observed less efficient processing of manual verbs in the presence of fast (compared to slow) concomitant actions. Likewise, motor-system damage distinctly affects processing of verbs that entail high (as opposed to low) motility [24] and fast (rather than slow) movements [23]. Taken together, then, present and previous results suggest that verbs conveying elevated motion intensity can hinder physical actions.

This finding is consistent with the HANDLE model [1]. Action verbs, in general, and manual verbs, in particular, increase activation in such networks [26] and modulate electrophysiological markers of response preparation and execution [2]. Accordingly, HANDLE posits that activity levels in manual motor networks are raised when processing action-laden words [1]. Such effects, we surmise, could be amplified by high motility verbs. Indeed, HANDLE posits that increased semantic demands lead to supra-threshold activation in hand-specific motor circuits, rendering them sub-optimally available for other processes, such as manual movements. (This phenomenon could also be influenced by predictive coding dynamics, as proposed below.) Thus, our findings support and extend a leading account of motor-language coupling.

Action planning and execution were also affected by semantic effector quantity. Both processes were facilitated by bimanual verbs relative to unimanual verbs and non-manual verbs, a pattern that held across motility levels for action planning and was restricted to high motility items for action execution. Crucially, since our task required keyboard typing, this result suggests that bimanual actions can be distinctly facilitated when verb meaning involves two hands. Previous studies showed that bimanual verbs, unlike unimanual verbs, engage both left and right motor regions [28] jointly implicated in bimanual movements [34, 35]. Insofar as word meanings reactivate their real-life sensorimotor correlates [36–38], we propose that bimanual verbs would prime bilateral manual action mechanisms.

This, too, aligns with predictions of HANDLE. Drawing on predictive coding tenets [39, 40], the model posits that manual verbs generate predictions that may or may not be met by subsequent manual actions. Here, bimanual verbs, unimanual verbs, and non-manual verbs would trigger embodied predictions of two-handed, one-handed, and non-manual actions, respectively. Accordingly, prediction errors would be minimized in the case of bimanual verbs, given that our task involved bimanual actions. Reduced error correction demands in these verbs’ forward models would involve a processing advantage, given that unimanual verbs and non-manual verbs would require further processing to reconcile their semantic expectations with the incongruence of a bimanual response. Indeed, latencies for bimanual verbs were similar to those of minimally motoric verbs during planning and shorter during execution. This attests to the magnitude of the observed facilitation, given that minimally motoric verbs comprise more abstract words that minimally engage motor networks [2, 9, 36, 41]–whereas the three other categories, all matched for concreteness and imageability, are known to engage sensorimotor circuits [26, 36, 42].

As stated earlier, effector quantity effects were not identical on FLL and WWL. The broad facilitation of bimanual verbs during planning became selective for high motility verbs during execution. This discrepancy might be related to the temporal dynamics of underlying neuronal activity. Specifically, both HANDLE (12) and an earlier simulation model [43] propose that effector congruency effects involve interference for early motor processes (occurring up to ≈400 ms post-stimulus onset) and facilitation for later motor processes (occurring up to ≈1000 ms seconds post-stimulus onset). This principle was corroborated by action planning (FLL) results, which showed facilitation for bimanual verbs before the 1000-ms mark. More particularly, HANDLE further posits that the duration of interference and facilitation effects can be substantially extended under increased semantic demands. This might explain why effector quantity effects during action planning (WWL) were limited to high motility items. As shown in Fig 1B, these items involved greater demands than low motility items. Such semantic exigency would extend the window of sub-threshold motor resonance, leading to more durable facilitation on congruent motoric responses (here, bimanual actions) [12]. Indeed, as proposed by Chersi and colleagues [43, p. 4], relevant neuronal pools “will respond faster or more slowly depending on whether their activation falls within the adaptation or the facilitation phase of previous pools.” In this sense, our study suggests that effector quantity and motility are interacting semantic factors that may jointly influence motor-language coupling dynamics. Yet, it remains unclear whether this pattern was mainly driven by reduced prediction errors, longer priming effects, or other dynamics related to specific neurotransmitters (NMDA, GABA, AMPA) contemplated by Chersi and colleagues [43]. This opens new avenues for novel neurocognitive studies on the topic.

Taken together, these results invite a more nuanced conceptualization of motor-language coupling in general, and of the HANDLE model in particular. While HANDLE captures numerous relevant aspects during processing of manual verbs at large, it lacks formulations for specific subsets thereof. In this sense, our study suggests that motor-language coupling effects are not only sensitive to effector specificity, but also, and more precisely, to the level of movement implied by the verb (motility) and to the match between the number of evoked and used effectors (effector quantity). Crucially, these two factors seem to have opposite behavioral correlates. We surmise that these discrepancies can be explained in predictive coding terms [29], on the assumption that response times increase as prediction errors increase. As regards motility, note that our task involved restricted movements, as typing requires moving one’s fingers while arms and other effectors remain static. Behavioral responses, then, would require correcting for more prediction errors in the case of high motility verbs, as their semantic prior of elevated motion would not be met by the low levels of motion that typing requires. Conversely, effector quantity involves varying levels of compatibility between verb-induced semantic predictions and response modality. Here, prediction errors would be reduced for bimanual verbs, as only these would match the bimanual nature of the behavioral response. Interestingly, minimally motoric verbs seem impervious to these effects, suggesting that only those categories that actually elicit sensorimotor resonance engage predictive coding dynamics during motor-language coupling. Looking forward, HANDLE should incorporate these notions in its descriptive and explanatory architecture, acknowledging the role of specific semantic distinctions and fine-grained predictive coding effects within the realm of hand-related words.

Our study also carries methodological implications. The motor-language coupling literature presents highly heterogeneous results, ranging from facilitation, to interference, to null effects, including distinct manifestations in action planning and execution stages. Current findings underscore verb motility and effector quantity as potential drivers of such discrepancies. Indeed, to the best of our knowledge, no single study in this line has controlled for the verbs’ action load or for the (mis)match between the number of evoked and employed effectors. Future designs could benefit from incorporating these factors in either their stimulus design or data analysis plans–together with other fine-grained variables, such as the speed implied by action verbs [22, 23].

## Limitations and avenues for further research

Despite its contributions, this study has a number of limitations. First, although our sample size was acceptably powered and larger than those of relevant antecedents [44, 45], it would be desirable to replicate the present experiment with more participants. Second, our study lacked a control condition comprised of (physical) unimanual actions, which would have motivated specific predictions for unimanual verbs. Future works should examine how the four verb categories tested here affect single-hand activities, such as pen writing [12]. Delving even deeper, new experiment could test whether motor-language coupling dynamics are sensitive to the (mis)match between the number of fingers evoked by verbs and used to respond. Third, our stimuli, analysis plan, and hypotheses were formulated by treating motility as a categorical variable (with high and low motility verbs). Yet, additional insights could be gained via different designs treating motility as a continuous variable, be it for covariance or correlational analyses. Fourth, note that out of 208 verbs in the study, 179 were transitive or ditransitive, mainly due to our focus on manual actions. Also, our stimuli were matched for nine psycholinguistic and six finger-distribution variables across eight conditions. These constraints preclude strict control of transitivity as a potential modulating factor. Yet, given its potential role in embodied dynamics, alternative paradigms could be devised that account for this variable. Fifth, despite its ecological properties, our paradigm employed relatively isolated stimuli. New investigations should include more context-rich materials, such as naturalistic narratives. This strategy would substantially enrich our understanding of motor-language coupling, while responding to recent calls for more ecological assessments of embodied language phenomena [41, 46–49]. Finally, all these efforts would benefit from preregistered designs involving multiple centers, as done in recent relevant work [8].

## Conclusions

This study showed that motor-language coupling is sensitive to verbs’ implied motility and effector quantity. Both variables affected the planning and execution stages of keyboard typing, as these were delayed by high motility verbs and facilitated by bimanual verbs–namely, verbs that evoked the same number of effectors used for responding. Such findings invite more refined accounts of how lexical semantics affects concomitant actions. New research on these and other sub-categories of manual verbs could enhance our understanding of effector-specific effects and embodied phenomena at large.

## Supporting information

S1 File (DOCX)

S2 File (DOCX)

S3 File (DOCX)
