# Supplementary material for: The embodied typist: Bimanual actions are modulated by words’ implied motility and number of evoked limbs
Source: PLoS One. 2023 Aug 10;18(8):e0289926. doi: 10.1371/journal.pone.0289926 (PMC10414656; doi:10.1371/journal.pone.0289926)
Supplement: S1 File — (DOCX) [file pone.0289926.s001.docx]

Supporting Information

The embodied typist: Bimanual actions are modulated

by words’ implied motility and number of evoked limbs

Katia Rolán^1,2^, Iván Sánchez-Borges^1^, Boris Kogan^3,4^, Enrique García-Marco^1,5^,

Carlos J. Álvarez^1^, Manuel de Vega^1^, Adolfo M. García^6,7,8,*^

^1^ Instituto Universitario de Neurociencia, Universidad de La Laguna, Spain

^2^ Laboratorio de Linguaxe e Cognición, Universidade de Vigo, Spain

^3^ Departamento de Filosofía, Facultad de Humanidades, Universidad Nacional de Mar del Plata, Buenos Aires, Argentina

^4^ Consejo Nacional de Investigaciones Científicas y Técnicas, Buenos Aires, Argentina

^5^ Departamento de Psicología Clínica y Experimental, Universidad de Huelva, Spain

^6^ Centro de Neurociencias Cognitivas, Universidad de San Andrés, Buenos Aires, Argentina

^7^ Global Brain Health Institute, University of California, San Francisco, USA

^8^ Departamento de Lingüística y Literatura, Facultad de Humanidades, Universidad de Santiago de Chile, Santiago, Chile

*** Corresponding author:**

E-mail: [adolfo.garcia@gbhi.org](mailto:adolfo.garcia@gbhi.org)

**Section 1. Power estimation details**

Using G*Power 3.1.9.6 (Erdfelder et al., 1996), we estimated power for a repeated measures 2x4 ANOVA, with motility (high, low) and effector quantity (bimanual, unimanual, non-manual, minimally motoric) as within-subject factors, and the following parameters: *p* = .05, *η*_p_**^2^** = .06, power = .80. Results indicated that a total sample size of 24 was enough to reach the estimated effects. Our actual sample size (*n* = 41) reaches a power of .97.

**Supplementary references**

Erdfelder, E., Faul, F., & Buchner, A. (1996). GPOWER: A general power analysis program. *Behavior Research Methods, Instruments & Computers*, *28*(1), 1-11. <https://doi.org/https://doi.org/10.3758/BF03203630>
