# Supplementary material for: The embodied typist: Bimanual actions are modulated by words’ implied motility and number of evoked limbs
Source: PLoS One. 2023 Aug 10;18(8):e0289926. doi: 10.1371/journal.pone.0289926 (PMC10414656; doi:10.1371/journal.pone.0289926)
Supplement: S2 File — (DOCX) [file pone.0289926.s002.docx]

Supporting Information

The embodied typist: Bimanual actions are modulated

by words’ implied motility and number of evoked limbs

Katia Rolán^1,2^, Iván Sánchez-Borges^1^, Boris Kogan^3,4^, Enrique García-Marco^1,5^,

Carlos J. Álvarez^1^, Manuel de Vega^1^, Adolfo M. García^6,7,8,*^

^1^ Instituto Universitario de Neurociencia, Universidad de La Laguna, Spain

^2^ Laboratorio de Linguaxe e Cognición, Universidade de Vigo, Spain

^3^ Departamento de Filosofía, Facultad de Humanidades, Universidad Nacional de Mar del Plata, Buenos Aires, Argentina

^4^ Consejo Nacional de Investigaciones Científicas y Técnicas, Buenos Aires, Argentina

^5^ Departamento de Psicología Clínica y Experimental, Universidad de Huelva, Spain

^6^ Centro de Neurociencias Cognitivas, Universidad de San Andrés, Buenos Aires, Argentina

^7^ Global Brain Health Institute, University of California, San Francisco, USA

^8^ Departamento de Lingüística y Literatura, Facultad de Humanidades, Universidad de Santiago de Chile, Santiago, Chile

*** Corresponding author:**

E-mail: [adolfo.garcia@gbhi.org](mailto:adolfo.garcia@gbhi.org)

**Section 2. Additional stimulus details**

**Table 1. Target verbs and approximate English translations.**

| **bimanual verbs** | | | **unimanual verbs** | | **non-manual verbs** | | | | **minimally motoric verbs** | |  |
| --- | --- | --- | --- | --- | --- | --- | --- | --- | --- | --- | --- |
| **Item** | **English translation** | **Item** | | **English translation** | | **Item** | **English translation** | **Item** | | **English translation** | |
| boxear | *box* | zurrar | | *baste* | | brincar | *jump* | espantar | | *scare* | |
| cavar | *dig* | apalear | | *beat* | | huir | *run away* | divertir | | *entertain* | |
| podar | *prune* | acuchillar | | *stab* | | convulsionar | *convulse* | cabrear | | *piss off* | |
| excavar | *excavate* | golpear | | *punch* | | competir | *compete* | enfadar | | *upset* | |
| arar | *plough* | serruchar | | *saw* | | patinar | *skate* | ultimar | | *finalise* | |
| destripar | *eviscerate* | pulir | | *polish* | | trepar | *climb* | humillar | | *humiliate* | |
| remar | *row* | perforar | | *drill* | | saltar | *jump* | aclarar | | *clarify* | |
| enterrar | *burry* | apedrear | | *stone* | | viajar | *travel* | acatar | | *abide* | |
| construir | *build* | mover | | *move* | | arrollar | *overwhelm* | obedecer | | *obey* | |
| talar | *chop down* | atizar | | *stoke* | | perseguir | *pursue* | desear | | *wish* | |
| desplumar | *pluck* | agitar | | *shake* | | embestir | *ram* | tardar | | *be late* | |
| batear | *bat* | pegar | | *paste* | | andar | *walk* | validar | | *validate* | |
| esculpir | *sculpt* | lijar | | *sand* | | patalear | *stamp* | prever | | *foresee* | |
| limpiar | *clean* | lanzar | | *launch* | | engullir | *swallow* | fingir | | *pretend* | |
| embalar | *pack* | extirpar | | *remove* | | pedalear | *pedal* | atrasar | | *delay* | |
| masajear | *massage* | arrojar | | *throw* | | rodar | *roll* | vacilar | | *vacillate* | |
| desmenuzar | *shred* | escarbar | | *scratch* | | venir | *come from* | afectar | | *affect* | |
| fregar | *scrub* | taladrar | | *drill* | | cojear | *limp* | traducir | | *translate* | |
| palpar | *palpate* | martillar | | *hammer* | | transitar | *transit* | opinar | | *opine* | |
| barrer | *sweep* | machacar | | *grind* | | arrodillar | *kneel* | compadecer | | *pity* | |
| teclear | *key in* | frotar | | *rub* | | temblar | *tremble* | abreviar | | *abbreviate* | |
| pescar | *fish* | manipular | | *manipulate* | | rebasar | *overrun* | valorar | | *value* | |
| duchar | *shower* | planchar | | *iron* | | traspasar | *transfer* | adorar | | *worship* | |
| cocinar | *cook* | abofetear | | *slap* | | vomitar | *vomit* | alegrar | | *brighten up* | |
| aprisionar | *shackle* | batir | | *beat* | | devorar | *devour* | adular | | *flatter* | |
| cachear | *search* | picar | | *sting* | | imitar | *imitate* | concienciar | | *raise awareness* | |
| arreglar | *fix* | moler | | *grind* | | acceder | *login* | afear | | *censor* | |
| empujar | *push* | hurgar | | *poke* | | pisar | *step* | imaginar | | *imagine* | |
| aflojar | *loosen* | abanicar | | *fan* | | vibrar | *vibrate* | comparar | | *compare* | |
| coser | *sew* | accionar | | *actuate* | | visitar | *visit* | tramar | | *plot* | |
| entrelazar | *entwine* | cepillar | | *brush* | | zampar | *scarf down* | acertar | | *get it right* | |
| zurcir | *darn* | girar | | *turn* | | tropezar | *stumble* | incumplir | | *breach* | |
| estirar | *stretch* | remover | | *remove* | | acelerar | *accelerate* | idear | | *devise* | |
| tender | *tend* | afeitar | | *shave* | | atropellar | *run over* | preferir | | *prefer* | |
| amarrar | *tie* | chasquear | | *snap* | | rebuscar | *rummage* | estimar | | *estimate* | |
| anudar | *knot* | apretar | | *tighten* | | besar | *kiss* | descuidar | | *neglect* | |
| aplaudir | *applaud* | raspar | | *scrape* | | adelantar | *advance* | valer | | *avail* | |
| abrazar | *cuddle* | exprimir | | *squeeze* | | vitorear | *cheer* | callar | | *shut up* | |
| enrollar | *roll* | transcribir | | *transcribe* | | acostar | *lay down* | memorizar | | *memorize* | |
| atar | *tie* | agarrar | | *grasp* | | masticar | *chew* | relajar | | *relax* | |
| abrochar | *buckle* | cortar | | *cut* | | animar | *animate* | disculpar | | *excuse* | |
| pelar | *peel* | colorear | | *colour* | | aclamar | *hail* | temer | | *fear* | |
| desatar | *unleash* | limar | | *file* | | morder | *bite* | adivinar | | *guess* | |
| tapar | *cover* | colgar | | *hang* | | chillar | *squeal* | acordar | | *agree* | |
| afilar | *sharpen* | escribir | | *write* | | beber | *drink* | asimilar | | *assimilate* | |
| tejer | *knit* | rascar | | *scratch* | | sollozar | *sob* | odiar | | *hate* | |
| arrugar | *wrinkle* | acariciar | | *caress* | | sonreír | *smile* | aburrir | | *bore* | |
| enlazar | *link* | borrar | | *delete* | | catar | *taste* | ignorar | | *ignore* | |
| colocar | place | anotar | | *annotate* | | gemir | *moan* | admirar | | *admire* | |
| acoplar | couple | inocular | | *inoculate* | | chupar | *suck* | dudar | | *doubt* | |
| doblar | fold | tachar | | *cross out* | | protestar | *protest* | envidiar | | *envy* | |
| desabrochar | undo | firmar | | *sign* | | silbar | *whistle* | intuir | | *intuit* | |

**Table 2. Statistical comparison of psycholinguistic properties between high and low motility verbs.**

|  | High vs. low motility bimanual verbs | High vs. low motility unimanual verbs | High vs. low motility  non-manual verbs | High vs. low  motility  minimally motoric verbs |
| --- | --- | --- | --- | --- |
| Average motility | **> 0.01** | **> 0.01** | **> 0.01** | **> 0.01** |
| Age of acquisition | 0.26 | 0.08 | 0.81 | 0.24 |
| Frequency | 0.58 | 0.43 | 0.46 | 0.76 |
| Letters | 0.88 | 0.64 | 0.33 | 0.85 |
| Syllables | 0.44 | 0.42 | 0.93 | 0.65 |
| Orthographic neighbors | 0.98 | 0.35 | 0.45 | 0.43 |
| Phonological neighbors | 0.88 | 0.29 | 0.35 | 0.47 |
| Familiarity | 0.64 | 0.17 | 0.08 | 0.20 |
| Imageability | 0.95 | 0.59 | 0.96 | 0.41 |
| Concreteness | 0.68 | 0.07 | 0.85 | 0.80 |
| Qwert | 0.94 | 0.83 | 0.54 | 0.38 |
| Asdfg | 0.57 | 0.43 | 0.72 | 0.28 |
| Zxcv | 0.71 | 0.30 | 0.15 | 0.67 |
| Yuiop | 0.44 | 0.54 | 0.32 | 0.07 |
| Hjkl | 0.05 | 0.08 | 0.59 | 0.57 |
| Bnm | 0.23 | 0.49 | 0.81 | 0.09 |

**Table 3. Descriptive statistics for each verb type.**

|  | Bimanual verbs | Unimanual verbs | Non-manual verbs | Minimally motoric verbs |
| --- | --- | --- | --- | --- |
| Average motility | 4.25 (0.97) | 4.09 (0.9) | 4.05 (1.04) | 1.82 (0.54) |
| AoA | 7.29 (2.12) | 7.39 (2.29) | 6.92 (2.58) | 7.81 (1.99) |
| Frequency | 3.15 (8.49) | 4.09 (8.83) | 6.27 (9.26) | 4.67 (5.01) |
| Letters | 6.85 (1.55) | 7.02 (1.49) | 7.06 (1.58) | 7.08 (1.31) |
| Syllables | 2.71 (0.64) | 2.79 (0.78) | 2.75 (0.74) | 2.92 (0.55) |
| Ort. neighbors | 5.5 (4.47) | 4.69 (3.31) | 4.21 (3.75) | 4.38 (3) |
| Phon. neighbors | 11.69 (8.81) | 9.83 (5.13) | 9.83 (6.34) | 9.79 (5.15) |
| Familiarity | 5.61 (0.71) | 5.62 (1.11) | 5.83 (0.6) | 5.58 (0.64) |
| Imageability | 5.27 (0.62) | 5.21 (0.93) | 5.11 (0.91) | 3.6 (0.61) |
| Concreteness | 4.71 (0.65) | 4.48 (0.87) | 4.76 (0.69) | 3.9 (0.57) |
| Qwert | 2.35 (1.17) | 2.33 (1.02) | 2.42 (1.07) | 2.27 (1.03) |
| Asdfg | 2 (0.97) | 1.88 (1) | 1.83 (1.02) | 2.12 (1.04) |
| Zxcv | 0.52 (0.75) | 0.58 (0.64) | 0.48 (0.58) | 0.56 (0.67) |
| Yuiop | 0.96 (1.05) | 1.23 (0.78) | 1.19 (0.89) | 1.23 (1.04) |
| Hjkl | 0.52 (0.58) | 0.54 (0.73) | 0.5 (0.75) | 0.35 (0.65) |
| Bnm | 0.5 (0.58) | 0.46 (0.61) | 0.62 (0.69) | 0.56 (0.64) |
| Means and standard deviations (in parentheses) for each Verb Type. Data extracted from Alonso et al. (2016), except for QWERTY keyboard areas and number of letters, which were counted ad-hoc. | | | | |

**Table 4. Statistical comparison of psycholinguistic properties across verb types.**

|  | Bimanual  vs unimanual | Bimanual  vs non-manual | Bimanual  vs min motoric | Unimanual  vs non-manual | Unimanual  vs min motoric | Non-manual  vs min motoric |
| --- | --- | --- | --- | --- | --- | --- |
| Motility | 0.38 | 0.31 | **> 0.01** | 0.83 | **> 0.01** | **> 0.01** |
| AoA | 0.83 | 0.42 | 0.20 | 0.33 | 0.31 | 0.05 |
| Frequency | 0.58 | 0.08 | 0.27 | 0.22 | 0.68 | 0.27 |
| Letters | 0.56 | 0.49 | 0.41 | 0.90 | 0.83 | 0.95 |
| Syllables | 0.58 | 0.78 | 0.07 | 0.80 | 0.31 | 0.18 |
| Ort. neigh. | 0.30 | 0.11 | 0.14 | 0.49 | 0.62 | 0.80 |
| Phon. neigh | 0.19 | 0.22 | 0.18 | 1.00 | 0.97 | 0.97 |
| Familiarity | 0.98 | 0.27 | 0.87 | 0.42 | 0.87 | 0.14 |
| Imageability | 0.83 | 0.52 | **> 0.01** | 0.70 | **> 0.01** | **> 0.01** |
| Concreteness | 0.37 | 0.79 | **> 0.01** | 0.22 | **0.01** | **> 0.01** |
| Qwert | 0.93 | 0.73 | 0.72 | 0.64 | 0.78 | 0.46 |
| Asdfg | 0.55 | 0.38 | 0.56 | 0.77 | 0.25 | 0.16 |
| Zxcv | 0.67 | 0.77 | 0.78 | 0.42 | 0.88 | 0.53 |
| Yuiop | 0.14 | 0.23 | 0.19 | 0.82 | 1.00 | 0.84 |
| Hjkl | 0.88 | 0.88 | 0.16 | 0.79 | 0.16 | 0.27 |
| Bnm | 0.74 | 0.36 | 0.63 | 0.23 | 0.43 | 0.66 |

**Table 5. Descriptive statistics of high motility verbs.**

|  | High motility bimanual verbs | High motility unimanual verbs | High motility non-manual verbs | High motility minimally motoric verbs |
| --- | --- | --- | --- | --- |
| Average motility | 5.1 (0.58) | 4.88 (0.48) | 4.92 (0.75) | 2.17 (0.58) |
| Age of acquisition | 7.64 (2.01) | 7.97 (1.89) | 7.01 (2.53) | 8.15 (2.12) |
| Frequency | 3.84 (11.79) | 3.07 (5.64) | 7.27 (10.6) | 4.89 (5.8) |
| Letters | 6.88 (1.64) | 7.12 (1.54) | 7.28 (1.77) | 7.04 (0.98) |
| Syllables | 2.64 (0.7) | 2.88 (0.83) | 2.76 (0.78) | 2.96 (0.45) |
| Orthographic neighbors | 5.48 (4.87) | 4.24 (3.07) | 3.8 (3.27) | 4.04 (2.21) |
| Phonological neighbors | 11.88 (10.16) | 9.04 (4.81) | 8.96 (5.26) | 9.24 (3.06) |
| Familiarity | 5.51 (0.72) | 5.15 (0.85) | 5.63 (0.64) | 5.39 (0.8) |
| Imageability | 5.26 (0.4) | 5.05 (1.04) | 5.11 (0.89) | 3.48 (0.44) |
| Concreteness | 4.79 (0.53) | 4.04 (1.03) | 4.74 (0.59) | 3.93 (0.59) |
| Qwert | 2.36 (1.22) | 2.36 (1.11) | 2.52 (0.96) | 2.4 (1) |
| Asdfg | 1.92 (0.86) | 2 (0.96) | 1.88 (1.2) | 2.28 (1.02) |
| Zxcv | 0.56 (0.82) | 0.48 (0.65) | 0.36 (0.57) | 0.6 (0.65) |
| Yuiop | 1.08 (1.26) | 1.16 (0.8) | 1.32 (0.99) | 0.96 (0.89) |
| Hjkl | 0.36 (0.49) | 0.72 (0.84) | 0.56 (0.71) | 0.4 (0.71) |
| Bnm | 0.6 (0.65) | 0.4 (0.58) | 0.64 (0.76) | 0.4 (0.5) |
| Means and standard deviations (in parentheses) for each high motility verbs. Data extracted from Alonso et al. (2016), except for QWERTY keyboard areas and number of letters, which were counted ad-hoc. | | | | |

**Table 6. Statistical comparison of psycholinguistic properties across high motility verbs.**

| **High motility** | Bimanual  vs unimanual | Bimanual  vs non-manual | Bimanual  vs min motoric | Unimanual  vs non-manual | Unimanual  vs min motoric | Non-manual  vs min motoric |
| --- | --- | --- | --- | --- | --- | --- |
| Motility | 0.16 | 0.32 | **> 0.01** | 0.88 | **> 0.01** | **> 0.01** |
| AoA | 0.84 | 0.21 | 0.44 | 0.14 | 0.54 | 0.05 |
| Frequency | 0.79 | 0.25 | 0.68 | 0.07 | 0.27 | 0.29 |
| Letters | 0.73 | 0.46 | 0.44 | 0.68 | 0.70 | 0.93 |
| Syllables | 0.37 | 0.57 | 0.06 | 0.73 | 0.54 | 0.27 |
| Ort. neigh. | 0.44 | 0.15 | 0.16 | 0.40 | 0.47 | 0.80 |
| Phon. Neigh | 0.30 | 0.20 | 0.18 | 0.73 | 0.71 | 0.97 |
| Familiarity | 0.41 | 0.74 | 0.75 | 0.17 | 0.56 | 0.44 |
| Imageability | 0.64 | 0.56 | **> 0.01** | 0.98 | **> 0.01** | **> 0.01** |
| Concreteness | 0.11 | 0.81 | **0.01** | 0.05 | 0.76 | **> 0.01** |
| Qwert | 0.91 | 0.61 | 0.90 | 0.51 | 0.79 | 0.67 |
| Asdfg | 0.88 | 0.79 | 0.24 | 0.70 | 0.33 | 0.22 |
| Zxcv | 0.59 | 0.18 | 0.74 | 0.36 | 0.34 | 0.07 |
| Yuiop | 0.60 | 0.33 | 1.00 | 0.54 | 0.53 | 0.26 |
| Hjkl | 0.11 | 0.37 | 1.00 | 0.48 | 0.16 | 0.43 |
| Bnm | 0.26 | 0.69 | 0.50 | 0.15 | 0.63 | 0.30 |

**Table 7. Descriptive statistics for low motility verbs.**

|  | Low motility bimanuals | Low motility unimanuals | Low motility non-manuals | Low motility minimally motoric |
| --- | --- | --- | --- | --- |
| Average motility | 3.46 (0.45) | 3.35 (0.46) | 3.24 (0.45) | 1.5 (0.18) |
| Age of acquisition | 6.97 (2.21) | 6.85 (2.53) | 6.84 (2.68) | 7.5 (1.84) |
| Frequency | 2.51 (3.5) | 5.03 (11.03) | 5.35 (7.91) | 4.46 (4.26) |
| Letters | 6.81 (1.49) | 6.93 (1.47) | 6.85 (1.38) | 7.11 (1.58) |
| Syllables | 2.78 (0.58) | 2.7 (0.72) | 2.74 (0.71) | 2.89 (0.64) |
| Orthographic neighbors | 5.52 (4.15) | 5.11 (3.51) | 4.59 (4.17) | 4.7 (3.6) |
| Phonological neighbors | 11.52 (7.54) | 10.56 (5.4) | 10.63 (7.2) | 10.3 (6.55) |
| Familiarity | 5.68 (0.72) | 5.9 (1.19) | 6.03 (0.5) | 5.72 (0.46) |
| Imageability | 5.28 (0.76) | 5.3 (0.89) | 5.1 (0.95) | 3.69 (0.71) |
| Concreteness | 4.66 (0.74) | 4.77 (0.64) | 4.79 (0.8) | 3.87 (0.58) |
| Qwert | 2.33 (1.14) | 2.3 (0.95) | 2.33 (1.18) | 2.15 (1.06) |
| Asdfg | 2.07 (1.07) | 1.78 (1.05) | 1.78 (0.85) | 1.96 (1.06) |
| Zxcv | 0.48 (0.7) | 0.67 (0.62) | 0.59 (0.57) | 0.52 (0.7) |
| Yuiop | 0.85 (0.82) | 1.3 (0.78) | 1.07 (0.78) | 1.48 (1.12) |
| Hjkl | 0.67 (0.62) | 0.37 (0.56) | 0.44 (0.8) | 0.3 (0.61) |
| Bnm | 0.41 (0.5) | 0.52 (0.64) | 0.59 (0.64) | 0.7 (0.72) |
| Means and standard deviations (in parentheses) for each low motility verbs. Data extracted from Alonso et al.. (2016), except for QWERTY keyboard areas and number of letters, which were counted ad-hoc. | | | | |

**Table 8.** **Statistical comparison of psycholinguistic properties across low motility verbs.**

| **Low motility** | Bimanual  vs unimanual | Bimanual  vs non-manual | Bimanual  vs min motoric | Unimanual  vs non-manual | Unimanual  vs min motoric | Non-manual  vs min motoric |
| --- | --- | --- | --- | --- | --- | --- |
| Motility | 0.38 | 0.08 | **> 0.01** | 0.38 | **> 0.01** | **> 0.01** |
| AoA | 0.90 | 0.92 | 0.27 | 0.99 | 0.41 | 0.42 |
| Frequency | 0.27 | 0.13 | 0.08 | 0.97 | 0.80 | 0.69 |
| Letters | 0.64 | 0.85 | 0.71 | 0.77 | 0.92 | 0.84 |
| Syllables | 0.83 | 0.83 | 0.51 | 1.00 | 0.42 | 0.42 |
| Ort. neigh. | 0.49 | 0.44 | 0.48 | 0.86 | 0.97 | 0.89 |
| Phon. Neigh | 0.44 | 0.67 | 0.62 | 0.78 | 0.81 | 0.95 |
| Familiarity | 0.60 | 0.12 | 0.87 | 0.61 | 0.60 | 0.05 |
| Imageability | 0.94 | 0.79 | **> 0.01** | 0.73 | **> 0.01** | **> 0.01** |
| Concreteness | 0.70 | 0.66 | **0.01** | 0.91 | **> 0.01** | **> 0.01** |
| Qwert | 1.00 | 1.00 | 0.54 | 1.00 | 0.50 | 0.55 |
| Asdfg | 0.37 | 0.33 | 0.80 | 1.00 | 0.52 | 0.47 |
| Zxcv | 0.20 | 0.26 | 1.00 | 0.82 | 0.15 | 0.20 |
| Yuiop | 0.09 | 0.49 | 0.05 | 0.29 | 0.56 | 0.15 |
| Hjkl | 0.11 | 0.34 | 0.05 | 0.69 | 0.64 | 0.45 |
| Bnm | 0.26 | 0.69 | 0.50 | 0.15 | 0.63 | 0.30 |
