# Supplementary material for: The embodied typist: Bimanual actions are modulated by words’ implied motility and number of evoked limbs
Source: PLoS One. 2023 Aug 10;18(8):e0289926. doi: 10.1371/journal.pone.0289926 (PMC10414656; doi:10.1371/journal.pone.0289926)
Supplement: S3 File — (DOCX) [file pone.0289926.s003.docx]

Supporting Information

The embodied typist: Bimanual actions are modulated

by words’ implied motility and number of evoked limbs

Katia Rolán^1,2^, Iván Sánchez-Borges^1^, Boris Kogan^3,4^, Enrique García-Marco^1,5^,

Carlos J. Álvarez^1^, Manuel de Vega^1^, Adolfo M. García^6,7,8,*^

^1^ Instituto Universitario de Neurociencia, Universidad de La Laguna, Spain

^2^ Laboratorio de Linguaxe e Cognición, Universidade de Vigo, Spain

^3^ Departamento de Filosofía, Facultad de Humanidades, Universidad Nacional de Mar del Plata, Buenos Aires, Argentina

^4^ Consejo Nacional de Investigaciones Científicas y Técnicas, Buenos Aires, Argentina

^5^ Departamento de Psicología Clínica y Experimental, Universidad de Huelva, Spain

^6^ Centro de Neurociencias Cognitivas, Universidad de San Andrés, Buenos Aires, Argentina

^7^ Global Brain Health Institute, University of California, San Francisco, USA

^8^ Departamento de Lingüística y Literatura, Facultad de Humanidades, Universidad de Santiago de Chile, Santiago, Chile

*** Corresponding author:**

E-mail: [adolfo.garcia@gbhi.org](mailto:adolfo.garcia@gbhi.org)

**Section 3. Additional results**

**Table 9. Full statistical details of the accuracy, FLL, and WWL analyses.**

| **Dependent variable** |  | ***F*** | | ***p-*value** | | $\boldsymbol{\eta}_{\mathbf{p}}^{\mathbf{2}}$ |
| --- | --- | --- | --- | --- | --- | --- |
| **Accuracy**  Motility |  | 1.53 | .22 | | .04 | |
| Verb Type |  | .27 | .84 | | .03 | |
| Motility* Verb Type |  | 1.31 | .27 | | .08 | |
| **FLL**  Motility |  | 21.77 | < .001 | | .35 | |
| Verb Type |  | 4.21 | < .01 | | .25 | |
| Motility* Verb Type |  | 1.18 | .32 | | .08 | |
| **WWL**  Motility |  | 70.71 | < .001 | | .64 | |
| Verb Type |  | 3.47 | .018 | | .24 | |
| Motility* Verb Type |  | 3.43 | .019 | | .23 | |
